# Tacrolimus Monotherapy is Safe in Immunologically Low-Risk Kidney Transplant Recipients: A Randomized-Controlled Pilot Study

**DOI:** 10.3389/ti.2022.10839

**Published:** 2022-10-24

**Authors:** Annelies E. de Weerd, Zainab Al Fatly, Marieken Boer-Verschragen, Judith A. Kal-van Gestel, Dave L. Roelen, Marjolein Dieterich, Michiel G. H. Betjes

**Affiliations:** ^1^ Department of Internal Medicine, University Medical Center Rotterdam, Erasmus MC Transplant Institute, Rotterdam, Netherlands; ^2^ Department of Immunology, HLA Laboratory, Leiden University Medical Center, Leiden, Netherlands

**Keywords:** kidney transplantation, infection, rejection, tacrolimus, immunosuppression reduction, mycophenolate mofetil

## Abstract

In this randomized-controlled pilot study, the feasibility and safety of tacrolimus monotherapy in immunologically low-risk kidney transplant recipients was evaluated [NTR4824, www.trialregister.nl]. Low immunological risk was defined as maximal 3 HLA mismatches and the absence of panel reactive antibodies. Six months after transplantation, recipients were randomized if eGFR >30 ml/min, proteinuria <50 mg protein/mmol creatinine, no biopsy-proven rejection after 3 months, and no lymphocyte depleting therapy given. Recipients were randomized to tacrolimus/mycophenolate mofetil (TAC/MMF) or to taper and discontinue MMF at month 9 (TACmono). 79 of the 121 recipients were randomized to either TACmono (*n* = 38) or TAC/MMF (*n* = 41). Mean recipient age was 59 years and 59% received a living donor transplant. The median follow-up was 62 months. After randomization, 3 TACmono and 4 TAC/MMF recipients experienced a biopsy-proven rejection. At 5 years follow-up, patient survival was 84% in TACmono versus 76% in TAC/MMF with death-censored graft survival of 97% for both groups and no differences in eGFR and proteinuria. Eleven TACmono recipients had an infectious episode versus 22 TAC/MMF recipients (*p* < 0.03). Donor-specific anti-HLA antibodies were not detected during follow-up in both groups. Tacrolimus monotherapy in selected immunologically low-risk kidney transplant recipients appears safe and reduces the number of infections.

## Graphical Abstract

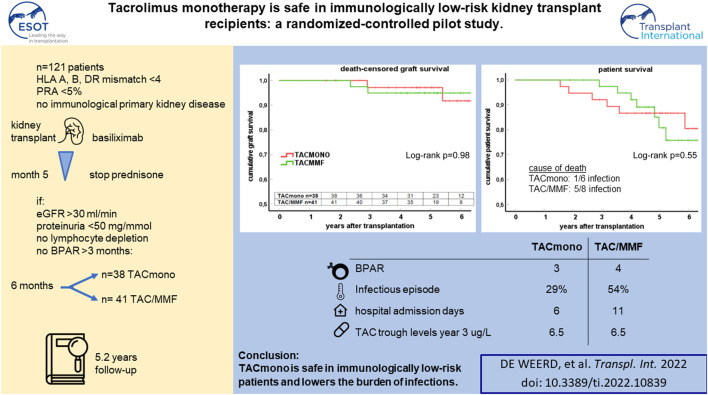

## Introduction

Kidney transplant recipients use life-long immunosuppression to prevent rejection and subsequent allograft loss. The only exceptions to this maintenance are recipients of a monozygotic twin donor kidney and the very rare recipients who demonstrate operational tolerance after discontinuation of immunosuppression. The most widely used combination of immune suppressive drugs consists of tacrolimus combined with mycophenolate mofetil in over 90% of recipients (1). As the risk for rejection is the highest in the first months after transplantation, induction therapy is administered at transplantation with a T-cell depleting agent in over 60% of recipients (1). Triple immunosuppression with steroids is used in the vast majority of recipients and over 60% use steroids after 1 year (2). Well-known side effects of immunosuppressive drugs are infection, malignancy and cardiovascular disease. The current SARS-CoV-2 pandemic for example has demonstrated that immunosuppression intensity puts solid organ transplant recipients at high risk of unfavorable outcomes (3). Immunosuppression, and specifically the use of mycophenolate mofetil results in worse vaccination responses (4, 5). Risk factors for rejection such as HLA-immunization and HLA mismatch can guide the choice for the initial immunosuppressive regimen with or without T cell depleting induction therapy. The risk for acute rejection declines rapidly after the first months after transplantation which follows the decrease in frequency of donor-specific alloreactive T cells (6). In accordance, most post-transplantation immune suppression protocols allow for a gradual stepdown in dose or number of immune suppressive drugs but there is currently a lack of reliable markers to guide weaning of immunosuppression.

Calcineurin inhibitors are the cornerstone of immunosuppressive regimens, and in previous weaning trials, discontinuation of tacrolimus led to a higher percentage of biopsy-proven acute rejections (BPAR) (7). Tacrolimus monotherapy has resulted in good outcomes when combined with depleting induction therapy (8). However, tacrolimus monotherapy after interleukin-2 receptor antibody induction in immunologically low-risk kidney transplant recipients (based on the frequency of interferon-γ expressing donor-specific alloreactive T cells, as described by Bestard et al.) increased acute rejection rates as compared to standard of care triple immunosuppression (9).

Based on these studies, it appears that tacrolimus monotherapy without T cell depletion early after kidney transplantation and tacrolimus withdrawal late after transplantation leads to a higher rejection incidence, even in immunologically low-risk patients. However, in older steroid withdrawal studies there is experience in tacrolimus monotherapy after non-depleting induction, demonstrating excellent graft outcomes despite a higher early rejection rate in the Atlas study (10). Tacrolimus monotherapy initiated at a later point in time after transplantation and without a prior severe rejection in the early post-transplantation period, may therefore still be an option and could reduce the incidence of adverse events in the long-term.

In this pilot study, lowering to tacrolimus monotherapy after non-depleting induction therapy was initiated 6 months after transplantation in immunologically low risk kidney transplant recipients, who were included at time of transplantation. The aim of this pilot study is to investigate the feasibility of a non-inferiority trial to determine the safety of tacrolimus monotherapy in immunologically low-risk kidney transplant recipients. Safety in terms of rejection, graft survival and donor-specific anti-HLA antibody (DSA) formation was assessed.

## Methods

### Study Design and Patients

We performed a randomized controlled, investigator-driven, open-label, single center pilot study from August 2014 till April 2018. Follow-up for data analysis was until March 2022. All recipients scheduled to receive either a deceased donor or a living donor kidney were screened for eligibility. Inclusion criteria were age 18 years and older, peak panel reactive antibodies (PRA) of <5% and HLA mismatches with the donor of ≤3. Re-transplantation was allowed when meeting these before mentioned inclusion criteria. Exclusion criteria were HLA-identical living-related transplantation, the presence of an immunological-mediated disease requiring (additional) immunosuppression, ABO-blood group incompatibility, a complement dependent cytotoxicity (CDC) or flowcytometry (FACS) positive cross-match, a combined liver/kidney or pancreas/kidney transplantation, the participation in another clinical trial and females of childbearing potential unwilling to use effective means of contraception. All recipients provided written informed consent before entry of the study during admission for kidney transplantation. This study is approved by the Medical Ethical Committee of the Erasmus Medical Center, conducted according to the Declaration of Helsinki and Declaration of Istanbul and registered in the Netherlands Trial Register [NTR4824, www.trialregister.nl].

### Randomization and Study Medication

All recipients were treated with the interleukin-2-receptor antibody (IL-2RAb) basiliximab, steroids, tacrolimus (TAC) and mycophenolate mofetil (MMF). Prednisolone 20 mg daily was tapered and discontinued at month 5 post-transplantation, target trough levels were for TAC 5-8 ug/L (once daily formulation Advagraf^R^) and MMF 1.5–3 mg/L from 3 months onwards in accordance with the standard protocol in our clinic. Recipients were included during admission. After a run-in period of 6 months they were randomized in a 1:1 ratio to either continue TAC and MMF (standard arm) or to halve their MMF dose at month 6 and discontinue MMF at month 9 while targeting for the same trough TAC levels (intervention arm). Randomization was carried out by an independent researcher with random allocation cards using computer-generated random numbers. Randomization criteria were eGFR >30 ml/min/1.73 m^2^ (CKD-EPI formula), proteinuria <0.5 mg protein/mmol creatinine in spot urine, freedom of biopsy-proven acute rejection (BPAR) from month three till six and the absence of lymphocyte depleting anti-rejection therapy. The full inclusion, exclusion and randomization criteria of this pilot study are described in Supplementary Table S1. Supplemental Figure S1 depicts the immunosuppressive regimens and trough levels.

### Study Objectives

The aim of this pilot study is to investigate the feasibility of a non-inferiority trial to determine the safety of tacrolimus monotherapy in immunologically low-risk kidney transplant recipients.

The feasibility objectives of this pilot study are:

1. Methodology: biopsy-proven acute rejection (BPAR)-rate
2. Process: willingness to participate in weaning study
3. Biological plausibility: (surrogate) parameters to assess treatment effect of less intensive immunosuppression:
  a. Biological plausibility of the benefit of discontinuing mycophenolate mofetil: hospital admission and infections.
  b. Secondary safety objectives: patient survival, death-censored graft survival, kidney allograft function and proteinuria.

No superiority or non-inferiority assessments are performed in this pilot study. We performed a post-hoc analysis on the number of symptomatic SARS-CoV-2 infections before the SARS-CoV-2 vaccination campaign till April 2021. This report analyzes these objectives, while detailed vaccination responses will be analyzed in a separate report [NL4824, www.trialregister.nl].

### Outcomes

BPAR was scored using the Banff-classification biopsies on *for cause* renal biopsies (11). Kidney function was measured with the CKD-EPI formula. Hospital admissions were defined as total number of (overnight) admissions in the transplant center till month 15 and in the referring hospitals thereafter. Total number of admitted days was also recorded. Infectious burden was defined as the sum of antibiotic use and CMV replication. Antibiotic use for at least three consecutive days was systematically recorded between month 6 and 15 (thereafter, due to referral to different hospitals, documentation of antibiotic use was more error prone). Serum CMV replication was measured by indication and 1 year after transplantation with polymerase-chain reaction (PCR). HLA antibodies were measured both 15 months and 4 years after transplantation with Luminex screening assay (Thermo Fisher Scientific, Waltham, MA). When present, HLA antibodies were further characterized with the Luminex single-antigen bead assay (12). SARS-CoV-2 infections were documented before April 2021 when the SARS-CoV-2 vaccination campaign commenced. A SARS-CoV-2 infection was scored when recipients were admitted to the hospital with positive polymerase chain reaction SARS-CoV-2 swab.

### Statistical Analysis

The sample size for this pilot study was calculated for the expected recruitment rate, one of the feasibility objectives. It was estimated that randomization of 80 patients would allow for a reasonable estimate of safety. Based on our historical data, it was expected that one third of recipients would not meet randomization criteria at month six because of rejection, low eGFR or proteinuria. It was estimated that if 120 patients gave consent out of 171 eligible patients, we could be 95% sure that the true consent rate will be between 63 and 77% (95% confidence interval of one proportion). The rough estimate therefore was that consent of at least 120 patients could determine the feasibility of the recruitment process and to allow for randomization of 80 patients. A planned interim analysis was performed after 40 patients had completed follow-up. The Data Safety Monitoring Board (DSMB) could advise to terminate the study if less than 15 recipients were included per year and when a difference in BPAR rate was observed between the treatment arms (“a sound clinical judgement that continuation of the study will harm recipients”). All patients who were randomly allocated to treatment were included in the analysis (intention-to-treat principle). Baseline characteristics were described according to distribution and type of data. We presented frequencies and proportions for categorical variables, means for normally distributed continuous variables and medians for continuous variables with a skewed distribution. Patient and graft survival was analyzed with the Kaplan-Meier log-rank test using SPSS version 21. Kidney function and proteinuria were analyzed with the Mann-Whitney-U test for differences between groups.

## Results

### Baseline Characteristics of Randomized Recipients

**FIGURE 1:**
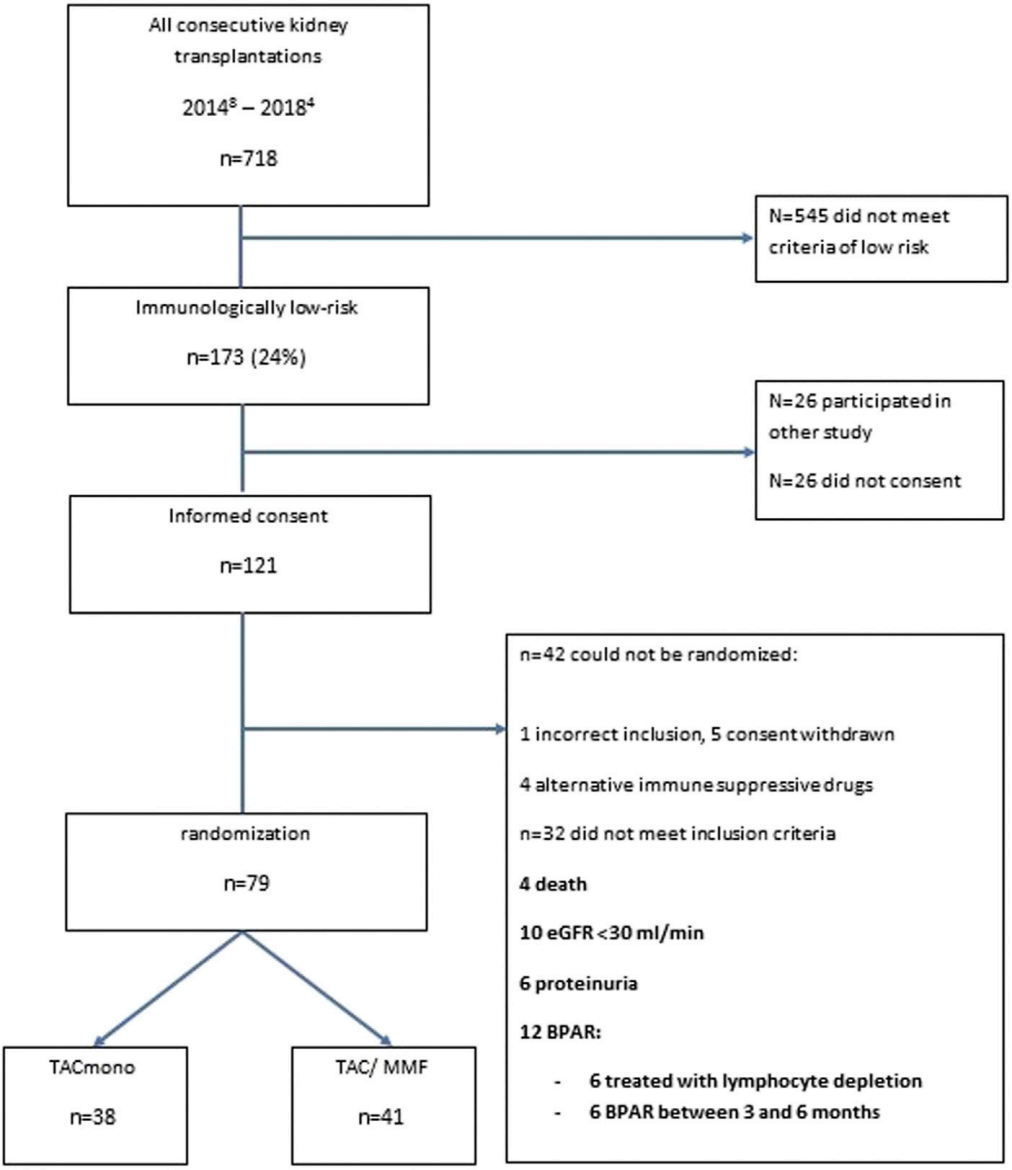
Flowchart of the selection of recipients included in the study.

Between August 2014 and April 2018, 718 adult kidney transplantations were performed. 170 (24%) of these procedures were in recipients who met our inclusion criteria of immunologically low risk recipients: 30% of deceased donor recipients *versus* 21% of living donor recipients. 147 recipients were counseled for this study of whom 121 gave written informed consent (consent rate 82%, Figure 1). After the run-in period of 6 months, 79 recipients could be randomized. Of the 42 non-randomized recipients, 12 had experienced BPAR of whom six had been treated with lymphocyte depleting anti-rejection treatment. Four of the non-randomized patients had been changed to an alternative immunosuppressive regimen: methotrexate for arthritis, continuation of prednisone with TAC trough level of 3 ug/L for arteriolar hyalinosis, and in two patients azathioprine for MMF-induced diarrhea.

Of the 79 recipients in study, 41 were randomized to standard TAC/MMF and 38 were randomized to the intervention TACmono. Mean age was 59.3 and 37% of recipients were 65 years of age and older (Table 1). The majority were male (73%), 59% received a living donor transplant, 3% received a second kidney transplant and 35% were transplanted pre-emptively. Diabetic nephropathy was the cause of end-stage kidney disease in 28%, hypertension in 22% and autosomal dominant polycystic kidney disease (ADPKD) in 10%. As steroid-responsive rejection in the first 3 months was not an exclusion criterion, 5 randomized recipients had experienced prior rejection. Of note is that four of these recipients were randomized to TACmono. Mean kidney function at month 6 was 55 ml/min with 17 mg protein/mmol creatinine in the urine. Six months after transplantation, mean TAC and MMF trough levels were 7.3 ug/L and 2.0 mg/L respectively at randomization.

**TABLE 1:** Baseline characteristics of kidney transplant recipients 6 months after transplantation, randomized to either tacrolimus monotherapy or dual tacrolimus and mycophenolate mofetil.

	TACmono *n* = 38	TAC/MMF *n* = 41
Age recipient (range)	59.6 (37–71)	59.0 (29–80)
Male (%)	76	71
Kidney disease (n)		
Diabetic nephropathy	11	11
Hypertension	7	10
ADPKD	4	4
Other	16	16
Age donor (SEM)	48.5 (2.3)	48.8 (2.7)
Total HLA mismatches (SEM)	2.1 (0.15)	2.4 (0.15)
PeakPRA (SEM)	2.4 (0.35)	3.4 (0.85)
Retransplantion (n)	1	1
Pre-emptive (%)	37	34
eGFR (CKD-EPI, ml/min/1.73 m^2^) (IQR)	53.8 (44–69)	50.4 (43–61)
Proteinuria (spot urine g/mol)	15.4 (1.5)	19.3 (6.0)
TAC trough ug/L (mean)	7.5 (0.43)	7.2 (0.38)
MMF trough mg/L (mean)	2.0 (0.18)	2.0 (0.18)
BPAR within 3 months after transplantation	4	1

ADPKD, autosomal-dominant polycystic kidney disease; HLA, human leucocyte antigen; IQR, interquartile range; MMF, mycophenolate mofetil; PRA, panel-reactive antibodies; BPAR, biopsy-proven acute rejection; SEM, standard error of the mean; TACmono, tacrolimus monotherapy.

### Patient and Graft Survival

**FIGURE 2:**
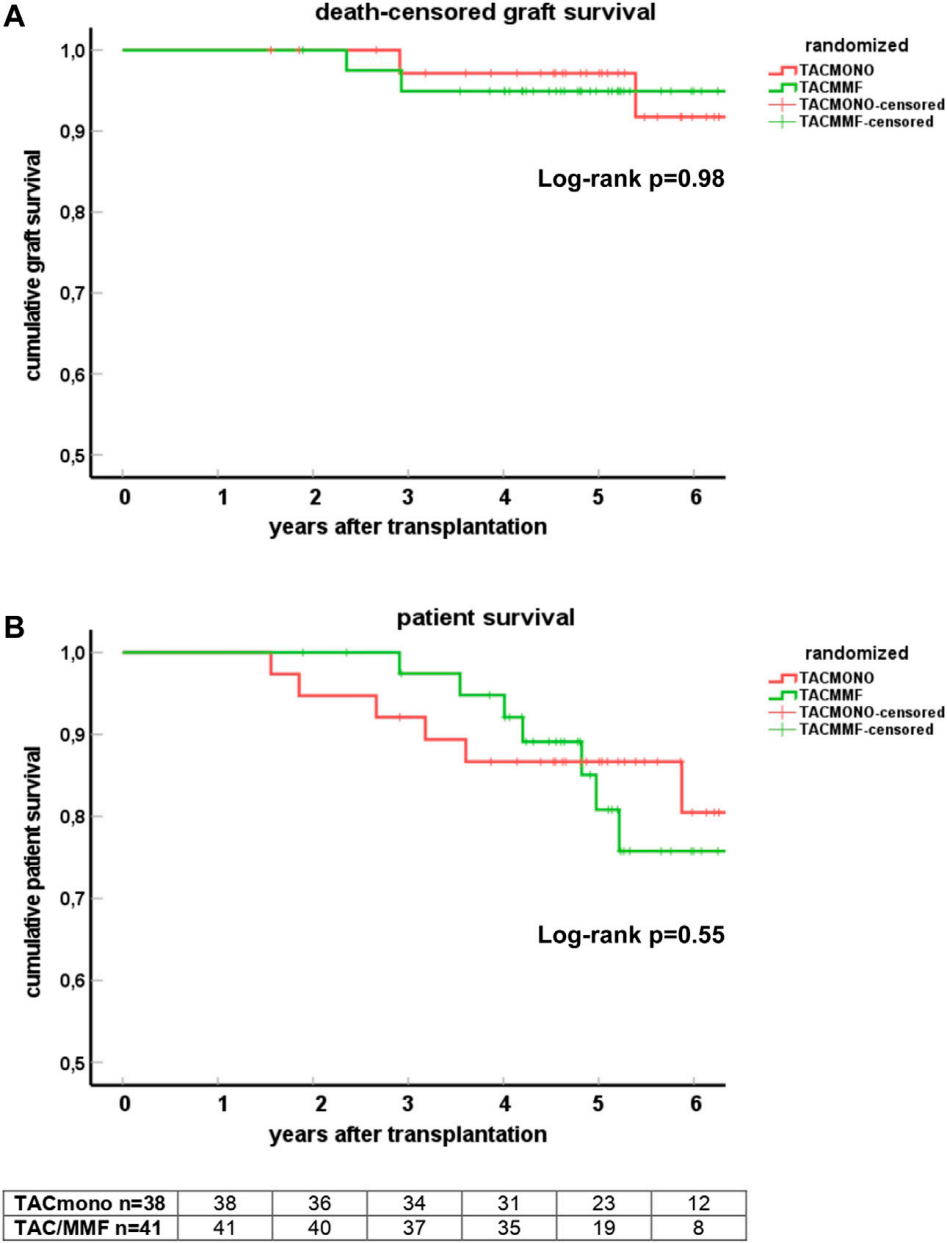
Death-censored graft survival **(A)** and patient survival **(B)** in kidney transplant recipients treated with either tacrolimus monotherapy or with standard tacrolimus/mycophenolate mofetil. Survival is shown by Kaplan-Meier cumulative survival curves. The group of recipients randomized to tacrolimus monotherapy (TACmono) or standard tacrolimus with mycophenolate mofetil (TAC/MMF) are shown as separate curves.

At 5 years follow-up, patient survival was 86% in the TACmono group *versus* 76% in the TAC/MMF group with 97% death-censored graft survival for both groups (log-rank test patient survival *p* = 0.55 and death-censored graft survival *p* = 0.98, Figure 2). Six TACmono and eight TAC/MMF patients died (Table 2). Causes of death were infection in one (diabetic ulcers), malignancy in two (stomach respectively pulmonary carcinoma), cardiovascular in two (heart failure, sudden cardiac death) and “other” (dementia) in one TACmono recipient. In contrast, causes of death in TAC/MMF recipients were sepsis in five (pneumonia, SARS-CoV-2 infection, decompensated hepatitis B, septic shock, urosepsis), malignancy in one (pulmonary carcinoma), cardiovascular in one (sudden cardiac death) and “other” in one (liver cirrhosis). Graft failure occurred in two TACmono recipients: one recipient lost his graft 35 months after transplantation due to chronic prostatitis with biopsy signs of urinary tract infection and borderline rejection (after experiencing Banff IA rejection at month 11). The other TACmono recipient lost his allograft 65 months after transplantation due to membranoproliferative glomerulonephritis (MPGN), which was interpreted as a probable recurrence of a previously undiagnosed MPGN. Two TAC/MMF recipients restarted dialysis, one recipient with mixed Banff IIA/antibody-mediated rejection due to non-adherence at month 28, and one recipient with Banff IIA vascular rejection at month 35.

**TABLE 2:** Cause of death in kidney transplant recipients, randomized to either tacrolimus monotherapy or dual tacrolimus and mycophenolate mofetil.

	TACmono	TAC/MMF
Number of recipients	38	41
Follow-up in months after transplantation (median and range)	64 (19–90)	60 (23–87)
Deceased at follow-up	6	8
Time to death after transplantation (median and range)	35 (19–70)	54 (42–86)
Cause of death		
Infection	1	5
Malignancy	2	1
Cardiovascular	2	1
Other	1	1
Graft loss other than death	2	2

TACmono, tacrolimus monotherapy; MMF, mycophenolate mofetil.

### Rejection Episodes After Randomization

During follow-up, seven recipients had experienced BPAR (Table 3). Three TACmono recipients experienced acute cellular rejection Banff IA 8, 10 and 11 months after transplantation (all within 3 months after discontinuation of MMF). These rejections were reversible with pulse methylprednisolone and thereafter their initial MMF dosage was restarted. One of these rejecting TACmono recipients had recurrent prostatitis and lost his graft 35 months after transplantation with biopsy signs of urinary tract infection and borderline rejection. Four TAC/MMF recipients experienced BPAR: one borderline cellular rejection at 9 months; one Banff IIA vascular rejection at 34 months; one histology of chronic-active antibody-mediated rejection, however without detectable DSA, after a CMV infection at 11 months; and one Banff IIA and mixed rejection (C4d positive, however without detectable DSA) due to non-adherence at 28 months after transplantation. Glomerulonephritis was diagnosed in two TACmono patients (with unknown primary kidney disease); membranoproliferative glomerulonephritis for which MMF was reinitiated and one IgA nephropathy with endocapillary proliferation which was treated with high dose steroids.

**TABLE 3:** Biopsy-proven acute rejections in kidney transplant recipients, randomized to either tacrolimus monotherapy or dual tacrolimus and mycophenolate mofetil.

	TACmono	TAC/MMF
n	38	41
follow-up in months (median, range)	64 (19–90)	60 (23–87)
BPAR	3	4
Type of rejection	Banff IA *n* = 0.3	Borderline rejection *n* = 1
		Histology of c-aABMR without DSA *n* = 1
		Banff IIA and mixed rejection without DSA *n* = 1
		Banff IIA *n* = 1

TAC, tacrolimus; MMF, mycophenolate mofetil; MPS, methylprednisolone; AMR, antibody-mediated rejection; caABMR, chronic-active antibody-mediated rejection; IVIG, intravenous immunoglobulins.

### Kidney Function, Proteinuria, DSA and TAC Trough Levels

**FIGURE 3:**
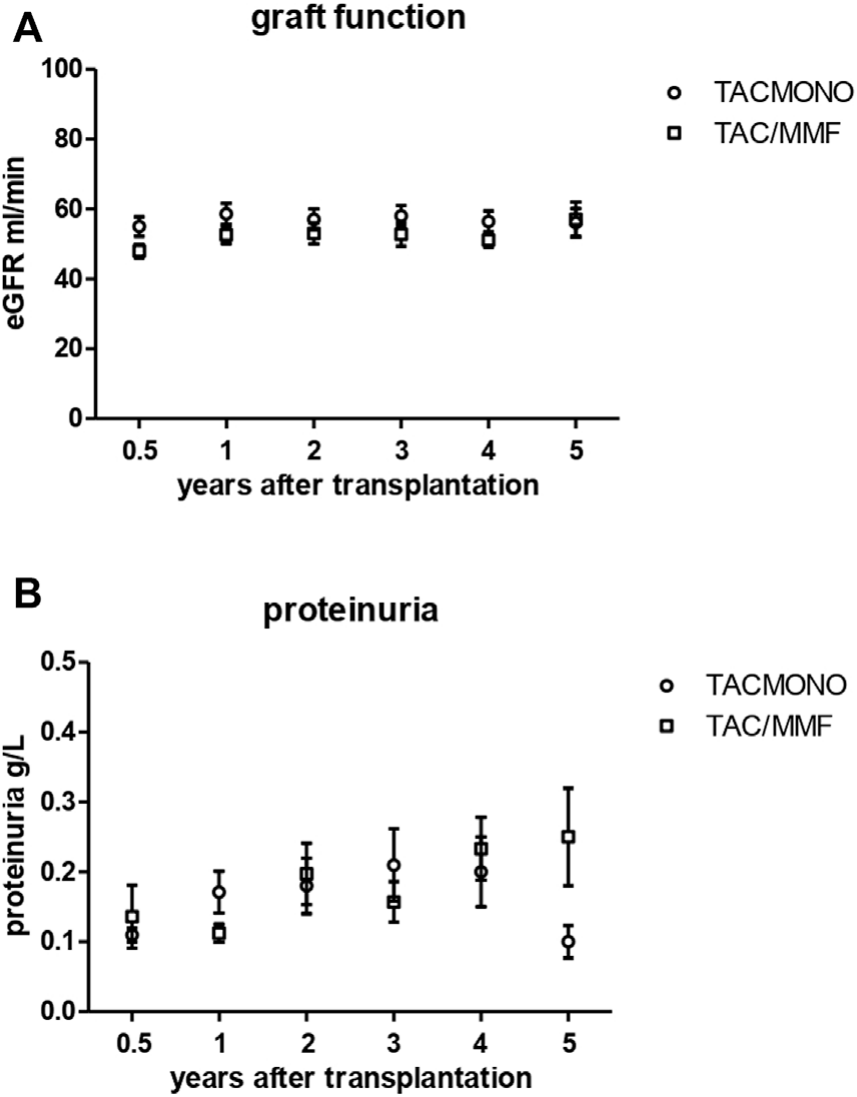
Kidney function **(A)** and proteinuria **(B)** in kidney transplant recipients treated with either tacrolimus monotherapy or with standard tacrolimus/mycophenolate mofetil. The eGFR (CKD-EPI formula) and proteinuria (in spot urine) is shown in the years after transplantation. The group of recipients randomized to tacrolimus monotherapy (TACmono) or standard tacrolimus with mycophenolate mofetil (TAC/MMF) are shown as separate data points.

At 1, 3 and 5 years of follow-up, kidney function and proteinuria were comparable between TACmono and TAC/MMF (Figure 3): eGFR was 58 vs. 52 ml/min at month 6 (*p* = 0.16) and 59 vs. 58 ml/min at year 3 (*p* = 0.98) in TACmono vs. TAC/MMF. Proteinuria was 0.15 *versus* 0.19 g/L at month 6 (*p* = 0.55) and 0.10 *versus* 0.25 g/L at year 5 (*p* = 0.53) in the TACmono *versus* TAC/MMF group. DSA were not detectable at time of transplantation. 15 months after transplantation Luminex screening did not reveal HLA-antibodies in randomized recipients. Four years after transplantation in only one recipient HLA-antibodies were detectable, which were non-donor HLA directed (in one TAC/MMF recipient after experiencing rejection). Tacrolimus trough levels 1 year, 3 years and 5 years posttransplant were 6.3, 6.5, 6.4 ug/L in TACmono vs. 6.2, 6.5 and 6.2 ug/L in TAC/MMF (Table 4).

**TABLE 4:** Hospital admissions, antibiotic use and CMV replication in kidney transplant recipients, randomized to either tacrolimus monotherapy or dual tacrolimus and mycophenolate mofetil.

	TACmono	TAC/MMF	*p-*value
n	38	41
follow-up in months (median, range)	64 (19–90)	60 (23–87)	0.25
Infectious burden	11 (29%)	22 (54%)	0.03
CMV viremia month 12	2 (5%)	4 (10%)	
Number of antibiotic treated recipients from month 6 till month 15	9 (24%)	14 (34%)	
Total of antibiotic courses	12	24	
Hospital admissions during follow-up			
Admissions (median, IQR)	1 (0–3.25)	2 (1,2,3)	0.32
Admitted days (median, IQR)	6 (9–17)	11 (3.5–24.5)	0.18
Tacrolimus trough levels (mean, SEM)			
month 6	7.5 (0.43)	7.2 (0.38)	0.63
year 1	6.3 (0.24)	6.2 (0.25)	0.72
year 2	6.7 (0.26)	6.4 (0.23)	0.76
year 3	6.5 (0.40)	6.5 (0.22)	0.10
year 4	6.4 (0.33)	6.8 (0.25)	0.56
year 5	6.4 (0.29)	6.2 (0.38)	0.41

IQR, interquartile range; SEM, standard error of the mean.

### Infectious Burden and Hospital Admissions After Randomization

Eleven TACmono *versus* 22 TAC/MMF recipients experienced infectious burden defined as antibiotic use and viral replication (*p* = 0.03, Table 4). Between 6 and 15 months after transplantation, infections needing antibiotics were recorded 12 times in 9 TACmono *versus* 24 times in 14 TAC/MMF recipients. Two TACmono recipients had detectable but asymptomatic CMV viral replication in serum 1 year after kidney transplantation. Four TAC/MMF recipients had an episode of symptomatic CMV viremia 1 year after transplantation, of which two developed CMV disease. The median number of hospital admissions was 1 (IQR 0–3.25) in TACmono *versus* 2 (IQR 1–3) in TAC/MMF (*p* = 0.32), with a total number of admitted days of 6 (IQR 0–17) *versus* 11 (IQR 3.5–24.5) in the TACmono vs. TAC/MMF, respectively (*p* = 0.18). At the onset of the SARS-CoV-2 pandemic in 2020 and before the vaccination campaign, two TACmono recipients and four TAC/MMF recipients were admitted to the hospital because of SARS-CoV-2 infection. One TAC/MMF recipient died of SARS-CoV-2 infection.

## Discussion

In this randomized controlled pilot study, tacrolimus monotherapy without mycophenolate mofetil appeared safe in immunological low-risk kidney transplant recipients and was associated with a lower rate of infections.

Rejection episodes were not increased in the tacrolimus monotherapy group. The few BPARS that occurred after randomization were easily reversible after steroid treatment. As one quarter of all consecutive kidney transplant recipients in our center met the immunological low-risk criteria as defined in this study, the identification of kidney transplant recipients who could benefit from less immunosuppression is relevant for a substantial portion of kidney transplant recipients. As our center has a large living donor program (65%) and a substantial number of immunized retransplant candidates, this portion of low risk recipients could likely be over one third of transplant recipients in other centers.

The introduction of calcineurin inhibitors (CNI) cyclosporin and tacrolimus in the standard immune suppressive regimen has dramatically reduced rejection incidence and subsequent increase graft survival in the short term (13). The potential nephrotoxicity of CNI has led to a number of studies aiming to lower or discontinue CNI in either recipients with a low immunological risk and/or long after transplantation when direct alloreactive T cell responses have declined. However, previous attempts to minimize calcineurin inhibitors early following kidney transplantations have shown discouraging high rejection rates in trials (7). Attempts to postpone weaning of CNI, after at least 4 years posttransplant, were also terminated prematurely: all five consecutive stable recipients who discontinued tacrolimus with or without steroids in Dugast et al. experienced either rejection or developed anti-HLA antibodies (14). Of note, the rationale for a CNI-free immune suppressive regimen in these studies was the potential nephrotoxicity of CNI. This side-effect was believed to be the most important cause for a relative lack in improvement of long-term graft patency observed after introduction of CNI. Most recent studies however, have pointed to chronic humoral rejection as the major cause for long-term graft failure and stress the importance of adequate trough levels and low intrapatient variability (15–19). Tacrolimus with trough levels above 5.0 ug/L have indeed been associated with improved graft survival (20, 21). A different strategy to minimize immunosuppression is therefore to maintain tacrolimus as the cornerstone of modern post kidney transplant immunosuppression, and to wean both steroids as well as mycophenolate mofetil. The recent CELLIMIN trial treated pre-transplant donor-specific IFN-γ T-cell ELISPOT (and DSA) negative recipients with tacrolimus monotherapy, but found high rejection rates comparable to ELISPOT-positive recipients treated with standard of care immunosuppression (9). Indeed, tacrolimus monotherapy without an increase in rejection incidence has only been achieved after depleting induction therapy (8, 10, 22). Despite higher early rejection rates in the Atlas study, basiliximab with tacrolimus monotherapy led to excellent graft outcomes (10).

The rationale behind the design of the current pilot study has taken these different observations into account and aimed for tacrolimus monotherapy in recipients with an *a priori* low risk for rejection and at a later point in time after transplantation. For extra safety, when immunological low risk recipients did have a severe rejection in the first 3 months or a late rejection after 3 months, poor graft function or proteinuria, they were not randomized. The results show that such an approach, pre-transplant immunological criteria combined with the clinical course in the first 6 months, identified recipients in whom weaning to tacrolimus monotherapy gave excellent outcomes. In addition, with an average tacrolimus trough level of 6 ng/L the graft function remained stable, no DSA developed and adherence to the once daily immunosuppressive regimen in the TACmono group was significantly better than in the TAC/MMF group (23). Another benefit TACmono recipients experienced, was improvement in diarrhea complaints after discontinuing MMF, as assessed with standardized questionnaires on gastro-intestinal symptoms (24).

The benefits of TACmono in terms of less adverse events is of course difficult to quantify in a pilot study. In our relatively small cohort, significantly less infections were noted and there was a trend towards less antibiotic use, and a trend towards less and shorter hospital admissions in recipients without MMF. Also, 5 out of 8 deceased TAC/MMF recipients died of an infectious cause of death, *versus* only 1 out of 6 deceased TACmono recipients. This pilot study however is not designed to dissect random errors from causality in graft survival differences and weaning immunosuppression. The goal of this pilot was to assess the feasibility of a larger weaning trial: the assuring comparable rejection rates in both groups, the willingness of recipients to lower their immunosuppression and the lower infection rates indicate that such a trial is worthwhile conducting. A strong indication for the benefit of tailored weaning was demonstrated by the severely hampered vaccination responses in TAC/MMF recipients in our cohort: in a substudy on SARS-CoV-2 vaccination only 7% of TACmono recipients were non-responders *versus* 38% non-responders in TAC/MMF (25).

There are a number of limitations of this study, apart from the obvious relative small cohort size as described above. The recipient age was 59 years and over one third of recipients was older than 65 years of age. This was a consequence of the exclusion of immunized recipients or those with an underlying immunological kidney disease. Elderly recipients age is associated with less rejection (26–29) and increased vulnerability for infections and these recipients may benefit specifically from minimized immune suppression. Whether the results can be generalized to younger recipients cannot be inferred from the current study.

This pilot approach with traditional immunological criteria as HLA matching and antibody screening can be implemented relatively easily. It is not known whether more granular immunological information such as HLA eplet matching can be more precise for identifying patients in whom tacrolimus monotherapy is safe.

To conclude, tacrolimus monotherapy from 9 months after transplantation appears safe in selected recipients with a proven low risk of acute rejection and is associated with a reduced risk of infection.

## Data Availability

The data that support the findings of this study are available from the corresponding author, upon reasonable request.

## Abbreviations

BPAR, biopsy-proven acute rejection; CDC, complement-dependent cytotoxicity; CNI, calcineurin inhibitors; DSA, donor-specific antibodies; DSMB, data safety monitoring board; eGFR, estimated glomerular filtration rate; FACS, flowcytometry; HLA, human leucocyte antigen; IL-2RAb, interleukin-2-receptor antibody; IQR, interquartile range; MPGN, membranoproliferative glomerulonephritis; MMF, mycophenolate mofetil; PCR, polymerase-chain reaction; PRA, panel-reactive antibodies; SARS-CoV-2, severe acute respiratory syndrome coronavirus 2; TAC, tacrolimus; TACmono, tacrolimus monotherapy.
