# Supplementary material for: Tacrolimus Monotherapy is Safe in Immunologically Low-Risk Kidney Transplant Recipients: A Randomized-Controlled Pilot Study
Source: Transpl Int. 2022 Oct 24;35:10839. doi: 10.3389/ti.2022.10839 (PMC9637544; doi:10.3389/ti.2022.10839)

SUPPLEMENTAL MATERIALS

Supplemental Table S1. Inclusion, exclusion and randomization criteria of 'Tacrolimus monotherapy in immunologically low-risk kidney transplant recipients: a randomized-controlled trial'. [NTR4824].

**Inclusion criteria at time of transplantation**	aged ≥18 years<br>peakPRA <5%<br>HLA mismatch <4<br>absence of immunological renal disease
**Exclusion criteria at time of transplantation**	ABO-incompatibility<br>CDC or FACS positive crossmatch<br>multi-organ transplantation<br>females of childbearing potential unwilling to use effective means of contraception
**Randomization criteria 6 months after transplantation**	eGFR >30 ml/min<br>proteinuria ≤ 50 mg/mmol in spot urine<br>no biopsy-proven rejection after 3 months<br>no lymphocyte depleting therapy

Supplemental Figure S1. Treatment schedule of the two randomized arms in the study 'Tacrolimus monotherapy in immunologically low-risk kidney transplant recipients: a randomized-controlled trial'. [NTR4824].

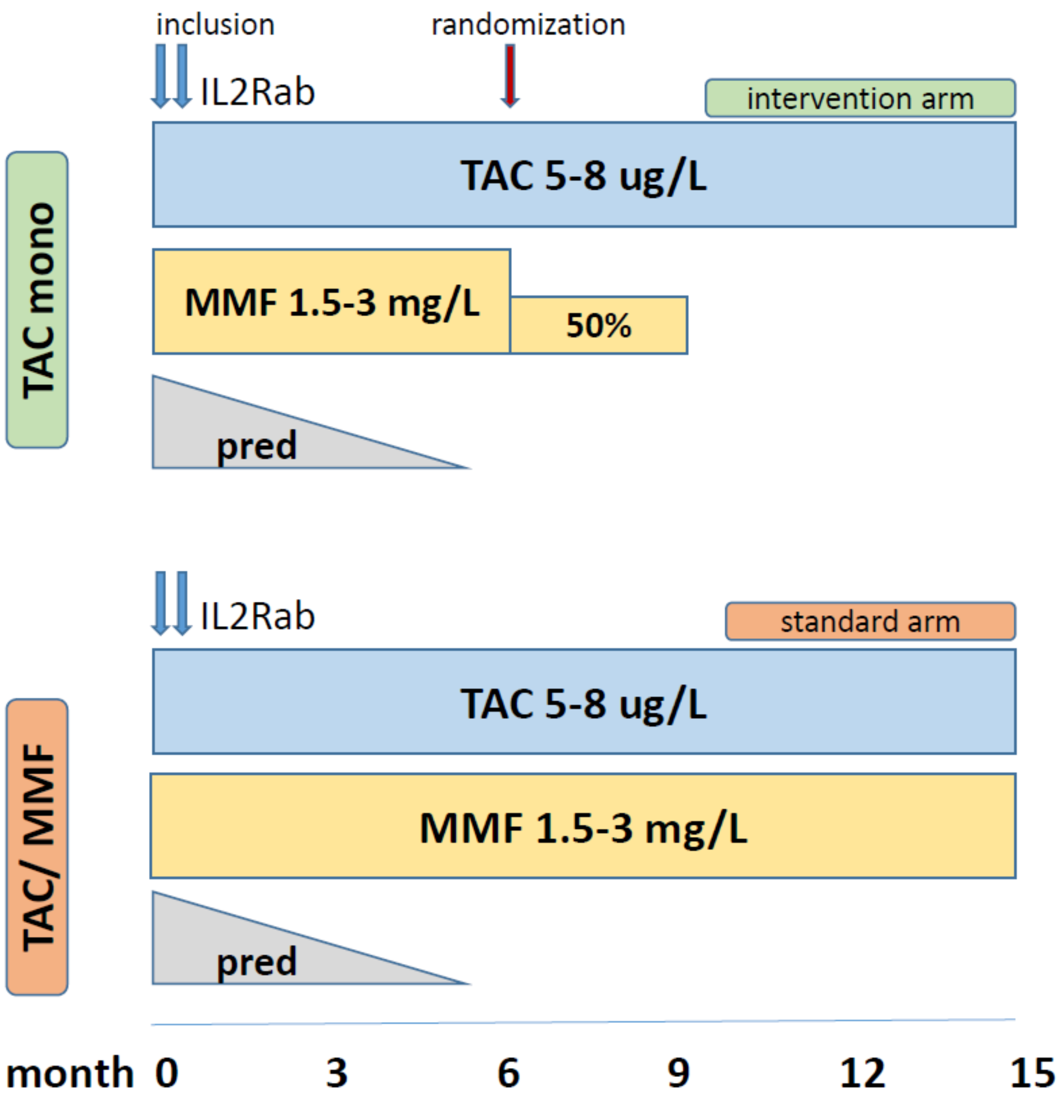

Supplement: Supplementary file 1 [file DataSheet1.PDF]
